# Supplementary material for: High Accuracy and Safety of Intraoperative CT-Guided Navigation for Transpedicular Screw Placement in Revision Spinal Surgery
Source: J Clin Med. 2022 Oct 2;11(19):5853. doi: 10.3390/jcm11195853 (PMC9573638; doi:10.3390/jcm11195853)
Supplement: Supplementary file 1 [file jcm-11-05853-s001.zip › jcm-1871323-supplementary.pdf]

Table S1. Demographic and Perioperative Data.

No	Age	Sex	Category of Etiology	Virgin Site Screws		Revision Site screws		Exchanged Screws
				Accepted screws	Unaccepted screws	Accepted screws	Unaccepted screws	
1	70	F	ASD	6	0	2	0	4
2	72	M	PLI	2	0	6	1(M)	0
3	92	F	PLI	0	0	6	0	0
4	74	F	Infection	4	0	0	0	6
5	76	F	MOS	4	0	6	0	0
6	55	M	ASD	6	0	0	0	6
7	73	F	PLI	6	0	6	1(L)	0
8	72	M	PLI	4	0	4	0	0
9	67	F	MOS	4	0	2	0	2
10	76	F	ASD	6	0	0	0	6
11	75	M	MOS	0	0	1	0	0
12	73	F	ASD	2	0	4	0	12
13	81	F	ASD	0	0	2	0	0
14	61	F	PLI	0	0	8	1(M)	0
15	55	M	PLI	2	0	6	0	0
16	47	M	PLI	0	0	6	0	0
17	56	M	ASD	8	0	0	0	4
18	76	F	PLI	2	0	6	1(L)	0
19	77	M	PLI	2	0	8	0	0
20	48	F	MOS	0	0	5	0	4
21	55	M	PLI	0	0	8	1(L)	4
22	66	F	ASD	3	1(M)	3	0	0
23	61	M	Infection	2	0	0	0	6
24	55	M	ASD	0	0	2	0	4
25	68	M	PLI	0	0	10	0	0
26	60	F	ASD	0	0	4	0	6
27	77	M	PLI	0	0	4	0	12
				63	1	109	5	76

Note. ASD: adjacent segment disease; MOS: malposition of previous transpedicular screw; PLI: post-laminectomy instability; (M): medial breach; (L): lateral breach; Revision site screws: Screws inserted at right over a previous laminectomy or posterolateral fusion field; Virgin site screws: Screws inserted at the site away from previous laminectomy or posterolateral fusion; Exchanged screws: Screws inserted to exchange preexisting screws with different style or brand screws through the previous trajectories.
